# Drug-Related Hospital Admissions and Associated Factors among Adults Admitted to Felege Hiwot Comprehensive and Specialized Hospital, North West Ethiopia

**DOI:** 10.1155/2022/6767488

**Published:** 2022-03-29

**Authors:** Leila Kenzu Kemal, Tigist Goshu Shewaga, Faisel Dula Sema

**Affiliations:** ^1^Department of Clinical Pharmacy, School of Pharmacy, College of Medicine and Health Sciences, University of Gondar, Gondar, Ethiopia; ^2^Department of Reproductive Health, Institute of Public Health, University of Gondar, Gondar, Ethiopia

## Abstract

**Background:**

A drug-related problem (DRP) is an event involving drug therapy that actually or potentially interferes with the desired therapeutic outcome. Drug-related hospital admission (DRHA) is hospitalization due to one or more DRPs.

**Objective:**

This study was aimed at assessing the prevalence of DRHA and factors associated with it among adults admitted to the internal medicine wards of Felege Hiwot Comprehensive Specialized Hospital.

**Methods:**

A prospective cross-sectional study was conducted using a previously validated tool, AT-HARM 10. Data were collected by two clinical pharmacists from July 1 to September 15, 2020. The data were entered into EpiData software (version 4.2.0.0) and then transported to Statistical Package for Social Sciences (SPSS®) software (version 24) (IBM Corporation) for analysis. Descriptive statistics were presented using frequency and proportion. Binary logistic regression was applied to identify factors associated with DRHAs with a 95% confidence level, and significance was declared at a *p* value <0.05.

**Results:**

The prevalence of DRHAs was 31.9% (95% CI = 27.7%–36.4%). From this, noncompliance (37.8%) (95% CI = 29.6–45.9), untreated indication (31.9%) (95% CI = 23.7–40), and adverse drug reaction (15.6%) (95% CI = 9.6–21.5) cause the majority of DRHAs. More than a quarter (28.8%) of all admissions were preventable. Most DRHAs were moderate (76.3) and preventable (80.7%). Lower to medium Charlson comorbidity index scores, longer duration of therapy, and not having health insurance were significantly associated with DRHAs.

**Conclusion:**

The prevalence of DRHAs was considerably high. Noncompliance, untreated indications, and adverse drug reactions were the commonest DRPs that caused DRHAs. Lower to medium Charlson comorbidity index scores, longer duration of therapy, and not having health insurance were significantly contributing factors of DRHAs. Therefore, all healthcare providers should prevent, identify, and resolve DRPs to decrease DRHAs in the hospital.

## 1. Introduction

Medication (drug) therapy is one of the forms of treatment intervention mostly used in any health practice setting. The aim is to maximize therapeutic benefits or optimize treatment outcomes [1]. This optimum outcome should be achieved by minimizing risk and by involving the patient in the decision-making process.

With the increased use of medicine, the occurrence of drug-related problems (DRPs) is being prevalent in both the inpatient and outpatient settings. DRP is defined as an event or circumstance involving drug therapy that actually or potentially interferes with desired health outcomes of the patient [2]. Based on Hepler and Strand, there are eight categories of DRPs [3]. These are untreated indications, improper drug selection, subtherapeutic dosage, failure to receive drugs, overdosage, adverse drug reactions (ADRs), drug interactions, and drug use without indication [3]. The other type of DRP that was not included under this classification was noncompliance [4].

Although the magnitude of DRPs varies with different diseases and study settings, its incidence reaches from 1.8 to 5.4 per patient [5, 6]. Moreover, the incidence of DRPs has shown a tendency to increase with time. For instance, the incidence of DRPs in ambulatory care units has increased from 9.1 to 16.9 per 1000 persons between 1995 and 2005 [7]. If unresolved, some of the DRPs are severe enough to cause serious negative health consequences that result in drug-related hospital admissions (DRHAs), which are hospitalizations due to one or more of the DRPs. However, a significant portion could be potentially preventable [8].

Although the occurrence of DRHAs may be unavoidable, their magnitude should be kept as low as possible with all necessary measures. However, previous reports showed that the prevalence of DRHAs ranges from 1.3% to 41.3% with an average of 15.4% in the globe [9] On the contrary, about one-third of these DRHAs would be preventable, and about 40% of them would be potentially preventable [9].

A systematic review of works of the literature showed ADR-related hospitalizations, which is only one part of DRHAs, cause 5.5% and 6.3% of hospitalizations in developing and developed countries, respectively [10]. Studies done in Ethiopia and South Africa also showed that the extent of ADR-related hospital admission was recorded as 10.4% and 8.4%, respectively [11, 12]. However, as far as the authors' literature review encompasses, there is no single study done on the extent of DRHAs caused by all types of DRPs.

Failure to prevent DRHAs through medication therapy optimization results in a cost beyond the purchase of prescribed medications as it includes additional medical costs of morbidity and mortality resulting from DRPs. The cost of prescription drug-related morbidity and mortality in the USA caused by nonoptimized medication therapies is about $528.4 billion [13]. It may not only cause an increased cost of drug use, but also harm patient like poor treatment outcome, impaired societal perception about medications, disability, and death [9]. In addition, patients may be exposed to hospital-acquired infections, which puts an additional burden on the patient, the patient's family, the healthcare system, and the community at large. It also adds a direct and indirect cost to the already fragile economy of developing countries like sub-Saharan African countries.

Despite this, to the best of our literature search and review, there is no study on DRHAs in Ethiopia. The only related available study is the one conducted in Jimma on ADR-related hospital admission, which is only one component of DRHAs [11]. Considering the risk and preventability of the DRHAs, this study was aimed at assessing the prevalence and associated factors of DRHAs at the internal medicine wards of Felege Hiwot Comprehensive and Specialized Hospital (FHCSH). It may contribute a piece of evidence for the promotion of rational drug use, prevention of antimicrobial resistance, and patient safety.

## 2. Materials and Methods

### 2.1. Study Design and Setting

A prospective cross-sectional study was conducted at the internal medicine wards of FHCSH in Bahir Dar, Amhara regional state from July 1 to September 15, 2020. Bahir Dar is located 576 km from the capital city of the country, Addis Ababa. The hospital is a tertiary care referral hospital with around 400 beds and 9 operating tables serving over 7 million people [14]. The internal medicine ward has 95 beds with different units, which are constituents, tetanus unit, tuberculosis (TB) unit, intensive care unit, psychiatry unit, and an oncology unit.

### 2.2. Study Population

The source populations were all patients who had admission to internal medicine wards of FHCSH. The study populations were all adult patients who had admission to internal medicine wards of FHCSH during the study period.

### 2.3. Inclusion and Exclusion Criteria

All adult patients (age ≥ 18 years) admitted to the internal medicine wards were included. Patients who have scheduled admission to internal medicine wards and patients with intentional medication poisonings were excluded.

## 3. Sample Size Determination and Sampling Technique

### 3.1. Sample Size Determination

The sample size was determined by using a single population proportion formula as follows: *n*=(*Z*^2^(*P*)(1 − *P*))/*d*^2^, where: *n* = sample size required, *d* = margin of error of 5% (*w* = 0.05), and *Z* = the degree of accuracy required (95% level of significance = 1.96). *P* = proportion (P) of 0.5(50%) was used to increase the study precision by getting a larger sample size. Although the proportion of DRHAs in previous studies is 1.3% to 41.3%, there is no study in a similar setting. *n*=((1.96)^2^(0.5)(1 − 0.5))/(0.05)^2^=384. After an adjustment was made for the nonresponse rate (10% contingency), the final sample size was 423 (384 + 38.4).

### 3.2. Sampling Technique

Data were collected on all patients who fulfilled the inclusion criteria within the study period. The sample size was used to guide the data collection period.

### 3.3. Variables of the Study

The dependent variable of the study was DRHAs, whereas the independent variables were the sociodemographic characteristics of the participants (age, sex, marital status, educational level, and place of residence), the status of health insurance, number of drugs prescribed, duration of drug therapy, Charlson comorbidity index (CCI) score, being on anti-retroviral therapy (ART), being on TB treatment, and kidney function.

### 3.4. Data Collection Instrument and Procedures

Both sociodemographic and clinical data were collected by two clinical pharmacists from patient charts and interviews within 48 hours of admission, by using structured questionnaires prepared based on previous studies (Supplementary Materials annex 1) [3, 10, 12, 15–17]. The patient interview was made to collect the sociodemographic characteristics of the participants, history of social drug use, and medication-related history, whereas the chief complaint, current diagnosis, history of present illness, and laboratory investigations were obtained from the patient charts. The comorbidity index was assessed by the CCI scores [15]. Cockcroft–Gault equation was used to calculate the estimated glomerular filtration rate [18].

A previously validated tool, AT-HARM 10, was used for the identification of DRHAs by clinical pharmacists based on their pharmacotherapy knowledge. For categorizing the DRPs, a classification method indicated within the tool was used [4, 19, 20]. When there is any ambiguity to put any DRP or DRHA under a specific category, it was presented to and decided based on the recommendation of at least two of the three clinical pharmacists. DRP was evaluated by using standard references including Ethiopian standard treatment guideline (STG 2014), Ethiopia National guideline for TB-Leprosy and DR_TB, Implementation Manual for DTG Rollout and ART Optimization, FY-2018 Ethiopia Malaria Operational Plan, Pharmacotherapy: A Pathophysiologic Approach, 10e, Harrison's principles of internal medicine 19e, up-to-date 2018, and Lexicomp drug interaction checker [21–27].

### 3.5. Data Quality Control Measures

The questionnaire was pretested on 5% (21 individuals) of the sample size at the University of Gondar Comprehensive and Specialized Hospital to assess the clarity of the questionnaire. The data collected during the pretest were not included in the analysis. Data collectors were pharmacists who have experience in clinical pharmacy service. The training was given for the data collectors before the start of data collection, and follow-up was done during the data collection period.

### 3.6. Data Processing and Analysis

The collected data were entered into EpiData version 4.2.0.0 software after checking their consistency and completeness and then transferred to the Statistical Package for Social Sciences (SPSS® (IBM Corporation)) software version 24 for analysis [28]. Descriptive statistics like frequency and proportion were used to analyze the sociodemographic and clinical characteristics of the participants. The dependent variable was dichotomized into DRHA and non-DRHA. The binary logistic regression analysis was done to identify factors associated with DRHAs. All independent variables which had a *p* value of less than 0.2 on the univariate analysis were used for the multivariate analysis. The normality of variables was checked using histogram and kurtosis. Model fitness was checked by using the Hosmer–Lemeshow goodness-of-fit test. All independent variables were checked for multicollinearity using variance inflation factor (VIF). All included variables to the multivariate analysis had a VIF between 1 and 1.8 [29]. All analyses were two-sided, a *p* value of less than 0.05 considered as statistical significance with a 95% level of confidence.

### 3.7. Operational Definition

The operational definitions of DRHA, the severity, and the preventability of DRHAs are provided in Table 1.

### 3.8. Ethical Consideration

Ethical clearance was obtained from the ethical review committee of the Department of Clinical Pharmacy, School of Pharmacy, College of Medicine and Health Science, the University of Gondar (ref. no. SOPs 069/2020). Then, permission was obtained from the medical director of FHCSH before starting the data collection. This study was conducted in accordance with the Declaration of Helsinki. Written informed consent was read and given to respondents to be signed, and respondents were assured that the information they gave would be kept confidential. They were also informed that they can refuse to participate in the study and have the full right to answer few or all questions, there is no way that their care would be affected due to their rejection of participation, and they can ask the data collector for anything they doubted about the study and/or the questions.

## 4. Results

### 4.1. Patient Demographics and Clinical Data

From 423 patients admitted to FHCSH, more than half (51.3%) were females. The median (interquartile range) age of the participants was 45 (32) years. Over two-thirds (69.3%) of the patients came from the rural part of the study area. More than half (60%) of the patients were illiterate (Table 2). The primary diagnosis of around one-fourth (25.8%) of the patients was a stroke; chronic kidney disease (stages 1 to 4) was also diagnosed in 4.5% of patients. Similarly, 7.6% and 9.9% of the patients were on ART and anti-TB treatment, respectively. About one-third (36.6%) of patients were on drug therapy for chronic diseases (Table 3).

### 4.2. Prevalence and Patterns of Drug-Related Hospital Admission

Among 423 patients admitted to the inpatient units, 31.9% (27.7%–36.4%) were DRHAs. More than a quarter (28.8%) of DRHAs were preventable. From this, 80.7% (73.3–87.4) were definitely preventable. Among the 135 DRHAs, the majority (76.3%) (68.9–83.7) were moderate. The three commonest DRHAs were due to noncompliance (12.1%), untreated indication (10.2%), and ADR (5%). The causality assessment for patients with ADR-related admission was done. Out of all ADR-related admission, 42.9% were definite and 57.1% were probable (Table 4).

Cardiovascular drugs account for 69 (51.1%) of DRHAs (Table 5). The most frequently involved drugs that cause ADR-related admissions were ART drugs (7), anti-TB drugs (5), warfarin (3), insulin (2), furosemide (1), phenytoin (1), vancomycin (1), and doxorubicin (1) (Table 6).

Others include the following: 31 of them have a different previous diagnosis (hypertension, cardiac disease, chronic kidney disease, and diabetes mellitus) because they have refused to start medications, and there was no written prescription. The rest of the drug-related hospital admissions were linked to phenytoin, haloperidol, and prednisolone.

### 4.3. Associated Factors with DRHAs

Patients with one up to five CCI scores, on longer duration of pharmacotherapy, and patients who do not have health insurance were significantly associated with DRHAs. Patients with lower CCI scores were significantly associated with DRHAs with an overall *p* value of 0.001. Patients with CCI score of 1–2 and 3–5 were 6.82 (2.39–19.46; *p* value = 0.0001) and 5.29 (1.55–17.99; *p* value = 0.008) times more likely to have DRHAs than patients without any comorbidity. The duration of drug therapy showed a significant association with DRHAs with an overall *p* value of 0.032. A one up to three-year duration of drug therapy has 7.45 (1.75–31.67; *p* value = 0.006) times more risk for DRHAs than being on no medication for chronic use. Although the overall *p* value showed the absence of significant difference for HIV status, being on ART tends to associate with DRHAs with a 6.56 (1.13–37.92) times more risk to DRHAs compared to HIV-negative individuals. Patients who do not have health insurance are 1.86 (1.06–3.25) times more likely to have DRHAs as compared to patients who have health insurance (Table 7).

## 5. Discussion

DRHA is hospitalization due to one or more DRPs. If these DRPs are not timely recognized and resolved, they may bring morbidity and mortality to the patient and cause a significant burden to the health care and the community. This study assessed the prevalence, patterns, and associated factors of DRHAs at the internal medicine wards of Felege Hiwot Referral Hospital, Bahir Dar, Northwest Ethiopia. It may contribute to the mitigation of the problem by providing scientific evidence for all concerned stakeholders and the scientific community.

In this study, the prevalence of DRHAs was 31.9% (27.7–36.4), which is considerably high. It is consistent with the study done in Malaysia (30.6%) and Norway Oslo (38%) [30, 31]. However, despite the similarity of the prevalence, studies on Malaysia and Norway were done on slightly different populations, so it should be interpreted with taking the necessary precautions. The study done in Malaysia was on adverse drug event (ADE)-related hospital admission, which means medical co-occurrence associated with drug use and may not have a causal relationship, and they also included intentional poisoning and drug misuse [31]. Whereas the study in Norway Oslo was done only among multimorbid patients [30], this is supported by the study done among old people with dementia in Sweden; the prevalence of DRHAs was 45.5% [32]. On the contrary, the prevalence of DRHAs in this study is much higher than studies done in south India (0.20%) [33], another study in India (3.3%) [34], Germany(0.7%) [35], Canada (24.1%) [36], Singapore 12.4% [37], and Saudi Arabia (4.5%) [38]. The higher prevalence in our study may be due to the use of the Helper and Strand comprehensive classification of DRHAs and also the tool's classification that we used for data collection, because these classifications are more inclusive and probably made the identification of more possible DRHA [3, 4, 19]. Generally, the variation seen between the studies may be due to the difference in the study settings, study population, and the definition used to categorize DRPs and DRHAs. Moreover, in sub-Saharan African countries like Ethiopia, due to the high number of illiteracies, low socioeconomic status, and poor healthcare setup, the management of chronic infectious and cardiovascular disease is difficult in the face of an increasing trend of cardiovascular disease and a wide prevalence of infectious diseases. From the base, due to the absence of proper and adequate diagnostic techniques, diagnosis and treatment are mainly based on the health professional subjective decision, leading to the use of many medications (polypharmacy) which imposes the risk of DRPs to the patient. On the other hand, most patients may not afford the medication cost, poorly understand the instruction given by the healthcare provider, fail to take the right dose of the medicine at the right time with the proper administration technique, be unable to complete or continue the intended treatment duration, and fail to visit health facilities for follow-up. Patients who failed to visit health facilities for their appointment may receive their medicines from community pharmacies without necessary investigations made for medication optimizations. Moreover, hesitancy, lack of awareness, and misconceptions of risky individuals and healthcare workers towards preventive measures may contribute to the burden in the era of COVID-19 pandemic [39, 40]. Considering the high prevalence of DRHAs, all necessary measures should be done for prevention, early identification, and timely resolution of DRPs. Having evidence of the burden of DRHA may help to draw the attention of all concerned bodies in this particular area. For taking the necessary action, understanding the patterns of DRHAs would have a great contribution, as well.

In this study, the proportion of patients admitted due to noncompliance was 51/423(12.1%) (95% CI = 9–14.9) which accounts 51/135 (37.8%) (95% CI = 29.6–45.9) of DRHAs. This result is consistent with the findings from the study done in Saudi Arabia (44.3%) [38]. It is also consistent with the study done in India, which reported that noncompliance accounted for 46.6% of DRHAs [34]. Similarly, the study about ADE-related hospital admission in Northern India showed that noncompliance accounts for 66% of DRHAs, which is much higher than the result of the current study [41]. They stated that the majority of patients who were taking conventional medicine switched to alternative medicine. The most mentioned reasons for noncompliance in this study were feeling well, drug costs, and trying nonconventional medicine. Patients from low socioeconomic status tend to discontinue their medication due to the cost of drugs [34]. Most patients may not understand that chronic diseases need lifelong therapy despite symptom resolution. Some patients try traditional medicine by discontinuing the prescribed medicine. Therefore, attention should be given to improving patient compliance with their medication. Provision of adequate information about their medication to patients through adequate counseling, inclining the care to patient-centeredness, promotion, and implementation of pharmaceutical care service, and integration of clinical pharmacists with the healthcare team may help to reduce the problem.

The second frequent cause of DRHAs in this study was untreated indication, 43/423(10.2%) (95% CI = 7.6–13.2) or 43/135(31.9%) (95% CI = 23.7–40). This is also a much higher figure than most studies. In two studies done in India and England, untreated indications were the fourth most frequent cause of DRHAs which accounted for 4.24% and 9%, respectively [34, 42]. In a study conducted in Hawaii, an untreated indication was the second frequent cause of DRHAs and accounts for 13.3% of DRHAs [43]. In a study conducted in Australia on ADE-related hospital admission, an untreated indication was the second frequent cause of hospital admission which accounts for 12.13% of ADE-related admission [44]. This may be because patients refuse to start medicines for confirmed chronic illness until they develop the severe symptoms and complications of the disease, psychologically and financially prepared, and due to preference to traditional medicine. These may contribute to the delay of treatment initiation, giving a chance for worsening of the disease symptoms and occurrence of complications leading to hospital admission. Provision of adequate counseling about the nature of chronic illnesses, execution of health insurance, awareness creation of the community on the issues of modern medicine, and trying to fulfill and advance diagnostic facilities may help reduce DRHAs due to untreated medical conditions.

ADR was the third most frequent DRP responsible for DRHAs. It accounts for 15.6% (21/135) (95% CI = 9.6–21.5) of DRHAs or 5% (21/423) (95% CI = 3.1–7.1) of all hospital admissions. It is almost consistent with the result of a study done in four hospitals of South Africa, accordingly which the prevalence of ADR-related admissions was 8.4% of all admissions [12], whereas it is slightly lower than the study done in Jimma, where the extent of ADR-related hospital admissions was 10.4% [10]. On the contrary, it is higher than the study done in France which reported a 3.6% incidence of ADR-related admissions. Even though ADR is the third commonest DRP after noncompliance and untreated indication in this study, it was the commonest type of DRP that cause DRHAs in the studies done in Canada and Saudi Arabia which account for 35.4% and 30.4% of DRHAs, respectively [34, 45]. However, it was the second frequent cause of DRHAs among other similar studies [17, 31, 34].

In this study, a causality assessment of ADRs was done using the Naranjo ADR probability scale. Out of 15.6% ADRs, 42.9% (9) were definite, and 57.1% (12) were probable. The drugs that cause ADR-related admissions out of the 21 ADR-related admissions were ART drugs (7), anti-TB drugs (5), warfarin (3), insulin (2), furosemide (1), phenytoin (1), vancomycin (1), and doxorubicin (1). Similarly, these drugs were mentioned as commonly involved drugs for ADE-related admission in South Africa [12]. The slight variation seen between the studies may be due to the difference in the study settings. Identifying the responsible medicines and the causality assessments may assist the necessary action required to tackle and prevent the problem. In addition, ADR reporting should be encouraged, promoted, and put into the habit of healthcare professionals.

Most of the DRHAs identified in this study were moderate to severe, 103 (76.3%) and 29 (21.5%), respectively. This finding is consistent with the result of previous studies done in India [34], Malaysia [31], and Canada [36], which reported that about three-fourths, more than two-thirds, and more than three-fourth were moderate DRHAs, respectively. This may be because patients come to the hospital when signaling signs and symptoms have occurred. Similarly, DRHAs with mild signs and symptoms may be either treated at an outpatient level or patients may not seek medical attention. Being most DRHAs moderate to severe may imply that a lot should be done at the prevention level of DRPs.

Generally, 28.8% (122/423) (25.1–32.9) of all admissions were potentially preventable. Out of 135 DRHAs, 109 (80.7%) of them were definitely preventable, and 13 (9.6%) of them were potentially preventable. This finding is consistent with the report from the study done in Hawaii in which 26% of DRHAs were preventable [43]. However, it is a little bit higher than the study was done in Australia, which reported that about 20.3% of admissions were preventable [46]. In this study, the majority of DRHAs were preventable, because they have arisen from noncompliance and untreated indications, which are categorized as preventable DRPs causing DRHAs. Although most of DRHAs are preventable, they are causing moderate to severe DRHAs which imposes a significant socioeconomic burden on the patients, the health care, the community, and the country. Therefore, a lot should be done to reduce the occurrence of preventable DRHAs. The healthcare team should be conscious of the nature of the problem, preventability, and act accordingly.

Since most DRPs could be resolved during the transition of care, medication reconciliation by clinical pharmacists during discharging of the patients may have a great contribution. The role of the pharmacist should come on to play by integrating clinical pharmacists into the healthcare team and advancing the dispensing system. All preventive measures being considered, intensive assessments of the contributing factor should also be done to help healthcare providers give special emphasis to people with a high risk of DRHAs.

Patients with CCIs score 1-2 and 3–5 are at increased risk of DRHAs than patients without any comorbidity. This is consistent with the findings from previous studies which reported that the increase in CCI score increases the odds ratio of DRHAs [12, 47–49]. In the study conducted in South Africa, a higher CCI score (>6) was associated with ADR-related hospital admission, this association may be due to the high prevalence of HIV in that area, and being acquired immunity deficiency syndrome (AIDS) patient scores 6 in CCI [12]. Whereas in our study, the higher CCIs score tends to lose its association with DRHAs, this is similar to a study done in Norway, in which a higher CCIs score was not associated with DRHAs. The relatively more frequent hospitalization of patients with higher CCI scores due to disease progression may mask the DRHAs.

Duration of drug therapy was associated with DRHAs with an overall *p* value of 0.032. One up to three years of drug therapy increases the risk of DRHAs by 7.45 (1.75–31.67) times than being without any medication for chronic use. This is in agreement with many previous studies which mentioned that the duration of drug therapy is a risk factor for noncompliance which is one of the primary components of DRHAs [50]. However, the association tends to disappear as the duration of therapy is greater than or equal to four years. This is consistent with a study done in Oklahoma, the USA, which reported that in cardiovascular patients the mean compliance ratio declines as the duration of therapy increased [51].

Although the overall *p* value showed the absence of significant difference for HIV status, being on ART tends to associate with DRHAs with a 6.56 (1.13–37.92) times more risk to DRHAs compared to HIV-negative individuals. This is in harmony with the study in South Africa on ADR-related hospital admission [12]. On the contrary, in two similar studies conducted in Jimma on ADR-related morbidity and predictors of ADR-related hospital admissions, being on ART was not associated with ADR-related hospital admission [10, 11]. The difference may be due to the inclusion of almost all DRPs responsible for DRHAs in this study unlike the studies in Jimma, which only consider ADR-related hospital admission.

Patients who do not have health insurance were more likely to have DRHAs as compared to patients who have health insurance. Patients with low socioeconomic status who do not have health insurance may discontinue their medication due to the inability to afford the cost of drugs and follow-ups. So, they may be unable to start or continue treatment. In two systematic reviews on DRHA due to nonadherence, the cost of medication was the commonest cause of noncompliance [52, 53]. Healthcare expenditures account for the larger amount of living cost and significantly affect patient compliance, especially on chronic patients who need lifelong treatment. A study done in India assessed whether the socioeconomic status of patients affects DRHAs, and being in low socioeconomic status was associated with DRHAs [34]. Therefore, expanding the coverage of health insurance for individuals with low socioeconomic status in Ethiopia may contribute to easing the problem.

## 6. Limitations

Being the first study by its type, it fills the information gap on the area and promotes the conduction of further multicenter studies in the country. However, despite all efforts made to assess the prevalence, patterns, and associated factors of DRHAs with quality data collection, not being a multicentered study may affect its generalizability. The other limitation of this study may be that it did not do the causality assessment, so the finding may need cautious interpretation. Although we have adopted and used a previously validated tool which was recommended for the identification of DRHAs [4, 19, 20], the population difference may slightly affect the study. Although we have calculated the sample size to determine the number of participants for the study, the confidence intervals are large and indicate that the sample size was not large enough with some outcome results. This may be an issue with the effect size being too large when determining the proportion of DRPs.

## 7. Conclusion

The prevalence of DRHAs among adult patients is considerably high. The top three most frequent causes of DRHAs were related to noncompliance to prescribed medication, untreated indications, and ADRs. Most of DRHAs were moderate to severe and preventable. Patients with comorbidity, longer duration of therapy, and who did not have health insurance have a high risk of DRHAs. Preventable DRHAs account for above one-fourth of all admissions. The considerably high prevalence of DRHAs can significantly affect patients and the healthcare system.

## 8. Recommendation

The hospital should give due emphasis to the reduction of the DRHAs by optimization of drug therapy by alerting all healthcare providers involved in patient care. The incorporation of clinical pharmacists and integrating them into the healthcare team may contribute a lot to the optimization of drug therapy through pharmaceutical care. Healthcare providers involved in patient care within the hospital should give special attention to patients with comorbidities, longer duration of therapy, and who do not have health insurance.

Interested researchers in the area could focus on patients with chronic disease and having multimorbid conditions. They also could focus on the assessment of the pharmaco-economic aspects of the problem.

## Figures and Tables

**Table 1:** Operation definitions.

	
DRHA	If the patient is admitted to the hospital due to one or more than one of the following potential DRPs: (1) untreated indications, (2) improper drug selection, (3) subtherapeutic dosage, (4) failure to receive drugs, (5) overdosage, (6) ADRs, (7) drug interactions, (8) drug use without indication, and (9) noncompliance [4, 16]
Non-DRHA	If (a) the admission is due to infection and previously undiagnosed disease, (b) the admission is due to the progression of the previously diagnosed disease, (c) the admission is due to physical trauma, substance intoxication, social circumstances, and allergies not related to medications [4, 16]
Definitely preventable	If the patient did not take a drug that is known to reduce or prevent the symptoms according to the prescriber directions, had a known allergy to the medication, had a disease for which the drug was contraindicated, and took a drug that was not indicated
Potentially preventable	If adequate monitoring prevents DRPs with reasonable time
Not preventable	If the drug event could not have been avoided by any reasonable means, or it was an unpredictable event in the course of treatment fully in accordance with good medical practice [3, 30]
Severity	Severity was considered as “mild,” if the laboratory abnormality or symptom was not requiring treatment; “moderate,” if the laboratory abnormality or symptom was requiring treatment or admission to hospital or resulting in nonpermanent disability, and “severe,” if abnormality or symptom that was life-threatening or resulted in a permanent disability or fatal [31, 32]
Duration of pharmacotherapy	The length of period that a patient stayed on drug treatment

DRHA: drug-related hospital admission.

**Table 2:** Sociodemographic characteristics of patients admitted to Felege Hiwot Comprehensive Specialized Hospital medical wards, from July 1 to September 15, 2020.

Characteristics		Frequency (%)
Sex	Male	206 (48.7)
	Female	217 (51.3)
Age	18–35	150 (35.5)
	36–64	171 (40.4.1)
	> = 65	102 (24.1)
Marital status	Single	63 (14.9)
	Married	287 (67.8)
	Divorced	31 (7.3)
	Widowed	42 (9.9)
Educational status	Illiterate	264 (62.4)
	Elementary school (1–8)	88 (20.8)
	High school (9–12)	41(9.7)
	Diploma and above	30 (7.1)
Area of residence	Rural	293 (69.3)
	Urban	130 (30.7)
Occupation	Retired	16 (3.8)
	Farmer	104 (24.6)
	Unemployed/housewife	220 (52.0)
	Employed/paid work	42 (9.9)
	Self-employed	37 (8.7)
	Other^*∗*^	4 (0.9)

^*∗*^Other includes prisoners, who run family's businesses.

**Table 3:** Clinical characteristics of patients admitted to Felege Hiwot Comprehensive Specialized Hospital medical wards, from July 1 to September 15, 2020.

Characteristics		Frequency (%)
Primary diagnosis	Stroke	109 (25.8)
	Cardiac disease	62 (14.7)
	Chronic kidney disease	15 (3.5)
	Diabetes mellitus	14 (3.3)
	Tuberculosis	14 (3.3)
	Human immune virus	24 (5.7)
	Deep vein thrombosis	24 (5.7)
	Chronic liver disease	14 (3.3)
	Pneumonia	15 (3.5)
	Meningitis	19 (4.5)
	Others^*∗∗*^	113 (26.7)
Chronic kidney disease	No CKD	380 (89.8)
	CKD stages 1–4	32 (7.6)
	ESRD	11 (2.6)
HIV status	Known on ART	30 (7.1)
	Unknown	225 (53.2)
	Tested negative	168 (39.7)
TB treatment	Yes	23 (5.4)
	No	400 (94.6)
Number of drugs before admission	1–2	67 (15.8)
	≥3	95 (22.5)
	None	261 (61.7)
Charlson comorbidity index	1–2	228 (53.9)
	3–5	57 (13.5)
	≥6	45 (10.6)
	None	93 (22)
Patients on drug therapy for chronic diseases	Yes	155 (36.6)
	No	268 (63.4)

^*∗∗*^Others include pancytopenia, anemia, immune thrombocytopenia, inflammatory bowel disease, metastasized cancers, and tetanus. CKD: chronic kidney disease, ART: anti-retroviral therapy, ESRD: end-stage renal disease, HIV: human immune virus, and TB: tuberculosis.

**Table 4:** Patterns of DRHAs among patients admitted to Felege Hiwot Referral Hospital medical wards, from July to September 15, 2020.

Characteristics	Drug-related hospital admissions	Frequency	Percent	95% confidence interval
Admission (*N* = 423)	DRHAs	135	31.9	27.7–36.4
	Non-DRHAs	288	68.1	63.8–72.3
Individual DRPs that cause drug-related hospital admission (*N* = 135)	Noncompliance^*∗*^	51	12.01	9–14.9
	Untreated indication	43	10.2	7.6–13.2
	Adverse drug reaction¥	21	5	3.1–7.1
	Subtherapeutic dosage	9	2.1	0.9–3.5
	Improper drug selection	8	1.9	0.7–3.3
	Drug interaction	1	0.23	0.0–0.7
	Over dosage	1	0.23	0.0–0.7
	Failure to receive drug	1	0.23	0.0–0.7
	Drug use without indication	0	0	
	Total	423	100	
Severity (*N* = 135)	Mild	3	2.2	(0.0–0.52)
	Moderate	103	76.3	68.9–83.7
	Severe	29	21.5	14.8–28.1
	Fatal	0	0	0
	Total	135	100	
Preventability (*N* = 135)	Defiantly preventable	109	80.7	73.3–87.4
	Potentially preventable	13	9.6	4.4–14.8
	Not preventable	13	9.6	5.2–14.8
	Total	135	100	

^*∗*^Common causes for noncompliance were feeling well (32), side effects (4), drug cost (4), due to pandemic (6), other reasons (5), other reasons include trying nonconventional medicine, family reason. Causality assessment was done using Naranjo ADR probability scale; 9 were definite and 12 were probable. DRPs: drug-related problems, DRHAs: drug-related hospital admissions.

**Table 5:** Class of drugs responsible for DRHAs among patients admitted to Felege Hiwot Referral Hospital medical wards, from July to September 15, 2020.

Class of drugs	Frequency	Percent
Antidiabetics	4	3.0
Anticoagulants	3	2.2
ART drugs	12	8.9
Antibiotics	1	0.7
Anti-TB drugs	5	3.7
Cardiovascular drugs	69	51.1
NSAIDs	1	0.7
Antineoplastic agents	2	1.5
Others∞	38	28.1
Total	135	100.0

ART: anti-retroviral therapy, DRHAs: drug-related hospital admissions, NSAIDs: nonsteroidal anti-inflammatory drugs, TB: tuberculosis.

**Table 6:** List of drugs implicated in ADR-related admissions among patients admitted to Felege Hiwot Referral Hospital medical wards, from July to September 15, 2020.

Drugs	Frequency
Insulin	2
Warfarin	3
Antiretroviral drugs	7
Vancomycin	1
Antituberculosis drugs	5
Phenytoin	1
Furosemide	1
Doxorubicin	1
Total	21

ADR: adverse drug reaction.

**Table 7:** Associated factors with DRHAs among patients admitted to Felege Hiwot Comprehensive Specialized Hospital medical ward from July to September 15, 2020.

Variables				COR (95% CI)	AOR (95% CI)	*p* value
		Yes	No			
Area of residence	Rural	84	209	0.62 (0.041–0.96)	1.06 (0.58–1.91)	0.844
	Urban	51	79	1	1	
Health insurance	Yes	63	157	1	1	
	No	72	131	1.36(0.9–2.06)	1.86 (1.06–3.25)	0.028^*∗*^
Chronic kidney disease						0.436
	No CKD	112	268	1	1	
	CKD	18	14	3.07 (1.49–6.40)	1.67 (0.40–6.83)	0.474
	ESRD	5	6	1.99(0.57–6.68)	2.58 (0.54–12.23)	0.231
HIV status						0.102
	On ART	17	13	2.91 (1.32–6.45)	6.56 (1.13–37.92)	0.035
	Unknown	66	159	0.92 (0.59–1.43)	0.97 (0.54–1.744)	0.941
	Tested negative	52	116	1	1	
Number of drugs						0.271
	1-2 drugs	44	23	14.73 (7.83–27.70)	3.09 (0.71–13.45)	0.131
	≥3	61	34	13.81 (7.84–24.33)	3.08 (0.76–12.33)	0.112
	None	30	231	1	1	
Charlson comorbidity index score						0.001^*∗∗*^
	None	5	88	1	1	
	1–2	81	147	9.69 (3.8–24.85)	6.82 (2.39–19.46)	0.0001^*∗∗*^
	3–5	29	28	18.22 (6.44–51.57)	5.29 (1.55–17.99)	0.008^*∗∗*^
	≥6	20	25	14.08(4.8° -41.29)	1.02 (0.16–6.24)	0.979
Duration of pharmacotherapy						0.032^*∗*^
	<1 years	22	17	10.26 (4.89–21.54)	3.23 (0.69–15.15)	0.136
	1–3 years	43	16	21.31 (10.67–42.5)	7.45 (1.75–31.67)	0.006^*∗∗*^
	>4 years	41	25	13.0 (6.92–24.4)	3.87 (0.92–16.2)	0.064
	None	29	230	1	1	

CKD: chronic kidney disease, ESRD: end-stage renal disease, ART: anti-retroviral therapy, HIV: human immune virus, ^*∗*^: *p* value <0.05, ^*∗∗*^: *p* value <0.001.

## Data Availability

All relevant data are in the manuscript. However, the minimal data underlying all the findings in the manuscript will be available upon request.

## Abbreviations

ADE: Adverse drug event
ADRs: Adverse drug reactions
AIDS: Acquired immunity deficiency syndrome
ART: Anti-retroviral therapy
CCI: Charlson comorbidity index
DRHAs: Drug-related hospital admissions
DRPs: Drug-related problems
FHCSH: Felege Hiwot Comprehensive Specialized Hospital
HIV: Human immunodeficiency virus
TB: Tuberculosis
VIF: Variance inflation factor.

## Supplementary Materials

Supplementary Materials Supplementary 1 (annex 1): a data collection questionnaire.
