# Supplementary material for: Drug-Related Hospital Admissions and Associated Factors among Adults Admitted to Felege Hiwot Comprehensive and Specialized Hospital, North West Ethiopia
Source: J Environ Public Health. 2022 Mar 29;2022:6767488. doi: 10.1155/2022/6767488 (PMC8983245; doi:10.1155/2022/6767488)
Supplement: Supplementary Materials — Supplementary 1 (annex 1): a data collection questionnaire. [file 6767488.f1.pdf]

Annex 1

Questionnaire

This study aimed at assessing the prevalence, patterns, and associated factors of drug-related Hospital admission among adult patients admitted to the medical wards of Felege Hiwot Comprehensive Specialized Hospital. It may contribute to the mitigation of the problem by providing a piece of scientific evidence on the magnitude, type, and associated factors of drug-related Hospital admission.

MRN____________________

1. **Socio-demographic information**

1.1. Age in years_________

1.2. Sex

A) Male
B) Female

1.3. Marital status

A) Single
B) Married
C) Divorced
D) Widowed

1.4. Educational level

A) Illiterate
B) Elementary school (grade 1-8)
C) High school (grade 9-12)
D) Diploma and above

1.5. Area of residence

A) Rural
B) Urban

1.6. Occupation

A) Retired
B) Farmer
C) Unemployed/house wife
D) Employee/paid work

E) Self-employed

F) If other specify______

2. Patient clinical data

2.1. Known allergic status

A) Yes

B) No

C) If yes (specify) ______

2.2. For how long the patient have been on drug therapy_____________

2.3. How many drugs prescribed before admission____________

2.4. Comorbid conditions

A) Heart failure

B) Diabetes mellitus

C) Hypertension

D) CKD

E) If other_______

F) None

2.5. HIV status

A) Known on ART

B) Unknown

C) Tested negative

2.6. On TB treatment

A) Yes

B) No

2.7. Chronic kidney disease

A) No CKD

B) CKD; stage_________

C) ESRD

2.8. Patient‘s assessment

2.9. Past medical history

2.10. Past medication history

2.11. Laboratory values and other diagnostic test

3. A-T-HARM 10 tool for identifying hospital admissions related to medications

questions	MRP category
U1. Was the admission caused by an infection or a previously undiagnosed disease (e.g. diabetes or heart failure) that is not medication-related?	n.a
U2. Was the admission caused by progression of a previously diagnosed disease that is not medication-related?	n.a
U3. Was the admission caused by physical trauma, substance intoxication, social circumstances or allergies that are not medication-related?	n.a
P4. Is it hinted or stated in the medical record that the admission was medication-related (including non-compliance)?	Any MRP category
P5. Might (side) effects of the medications the patient was taking (prescribed or not prescribed) prior to hospitalization have caused the admission (including over-treatment)?	5. Over dosage<br>6. Adverse drug reaction<br>8. Drug use without indication
P6. Are there abnormal laboratory results or vital signs that could be medication-related and might have caused the admission	2. Improper drug selection<br>5. Over dosage
P7. Was there any drug-drug interaction or drug-disease interaction (i.e. a contraindication) that might have caused the admission?	2. Improper drug selection<br>7. Drug interaction
P8. Did the patient have any previously diagnosed untreated or sub-optimally treated (e.g. dose too low) indications that might have caused the admission?	1. Untreated indication<br>2. Improper drug selection<br>Sub therapeutic dosage

P9. Was the patient admitted because of a problem with the dosage form or pharmaceutical formulation (i.e. failure to receive the medication)?	4. Failure to receive drug
P10. Is the cause of the admission a response to cessation or withdrawal of medication therapy?	6. Adverse drug reaction

4. If DRHA is possibly

4.1. Class of drugs likely responsible for DRHA

A) Anti- diabetics
B) Anti-coagulants
C) ART drugs
D) Antibiotics
E) Anti TB drugs
F) Cardiovascular drugs
G) NSAIDS
H) Antineoplastic
I) If other specify__

4.2. Mention if particular drug/s is responsible for DRHA ___________________

4.3. Severities of DRHA

A) Mild
B) Moderate
C) Severe

4.4. Preventability of DRHA

A) Definitely preventable
B) Potentially preventable
C) Not preventable

5. **Patient interview questionnaire for patients who have been on drug therapy before admission.**

5.1. Do you have regular follow-up?

A) Yes B) No

5.2 How do you get your mediation and treatments?

A) Free B) Paid

5.3 Do you have problems with remembering your medication regimen?

A) Yes B) No

5.4 Do you think your medication(s) helps to improve your health?

A) Yes B) No

5.5 Did you discontinue your medication without telling to your Doctor?

A) Yes B) No

5.6 If there is drug discontinuation, what is the cause

A) Drug cost
B) Side effect
C) Feeling well
D) Other reason
E) Thinking medications may not work

5.7 Do you have medications other than the prescribed medications?

A) OTC drugs
B) CAM
C) Prescription drugs
D) None

6. Charlson comorbidity index (CCI)

Comorbidity	Score
Prior myocardial infraction	1
Congestive heart failure	1
Peripheral vascular disease	1
Cerebrovascular disease	1
Dementia	1
Chronic pulmonary disease	1
Rheumatologic disease	1
Peptic ulcer disease	1
Mild liver disease	1
Diabetes	1
Cerebrovascular(hemiplegia) event	2
Moderate-to-severe renal disease	2
Diabetes with chronic complications	2
Cancer without metastasis	2
Leukemia	2
Lymphoma	2
Moderate or severe liver disease	3
Metastatic solid tumor	6
Acquired immune-deficiency	6
**Charlson comorbidity index CCI score(total):**	

7. Adverse Drug Reaction Probability Scale (if ADR related admission)

Question	Yes	No	Do Not Know	Score
1. Are there previous conclusive reports on this reaction?	+1	0	0	
2. Did the adverse event appear after the suspected drug was administered?	+2	-1	0	
3. Did the adverse event improve when the drug was discontinued or a specific antagonist was administered?	+1	0	0	
4. Did the adverse event reappear when the drug was re-administered?	+2	-1	0	
5. Are there alternative causes that could on their own have caused the reaction?	-1	+2	0	
6. Did the reaction reappear when a placebo was given?	-1	+1	0	
7. Was the drug detected in blood or other fluids in concentrations known to be toxic?	+1	0	0	
8. Was the reaction more severe when the dose was increased or less severe when the dose was decreased?	+1	0	0	
9. Did the patient have a similar reaction to the same or similar drugs in any previous exposure?	+1	0	0	
10. Was the adverse event confirmed by any objective evidence?	+1	0	0	
**Total Score:**				

Naranjo Algorithm - ADR Probability Scale

Score	Interpretation of Scores
**Total Score≥9**	**Definite**. The reaction (1) followed a reasonable temporal sequence after a drug or in which a toxic drug level had been established in body fluids or tissues, (2) followed a recognized response to the suspected drug, and (3) was confirmed by improvement on withdrawing the drug and reappeared on re exposure.
**Total Score5 to 8**	**Probable**. The reaction (1) followed a reasonable temporal sequence after a drug, (2) followed a recognized response to the suspected drug, (3) was confirmed by withdrawal but not by exposure to the drug, and (4) could not be reasonably explained by the known characteristics of the patient's clinical state.
**Total Score1 to 4**	**Possible**. The reaction (1) followed a temporal sequence after a drug, (2) possibly followed a recognized pattern to the suspected drug, and (3) could be explained by characteristics of the patient's disease.
**Total Score≤0**	**Doubtful**. The reaction was likely related to factors other than a drug.
